# Neurotransplantation of stem cells genetically modified to express human dopamine transporter reduces alcohol consumption

**DOI:** 10.1186/scrt36

**Published:** 2010-12-01

**Authors:** Tom N Grammatopoulos, Susan M Jones, Masami Yoshimura, Brian R Hoover, Mita Das, Evan Y Snyder, Gaynor A Larson, Nancy R Zahniser, Boris Tabakoff, W Michael Zawada

**Affiliations:** 1 Department of Medicine, University of Colorado Denver, 12800 East 19th Avenue, Aurora, CO 80045, USA; 2 Department of Pharmacology, University of Colorado Denver, 12800 East 19th Avenue, Aurora, CO 80045, USA; 3 Division of Pharmaceutical Sciences, School of Pharmacy, University of Wyoming, 1000 East University Avenue, Laramie, WY 82071, USA; 4 Neuroscience Program, University of Wyoming, 1000 East University Avenue, Laramie, WY 82071, USA; 5 The Sanford Burnham Medical Research Institute, 10901 North Torrey Pines Road, La Jolla, CA 92037, USA

## Abstract

**Introduction:**

Regulated neurotransmitter actions in the mammalian central nervous system determine brain function and control peripheral organs and behavior. Although drug-seeking behaviors, including alcohol consumption, depend on central neurotransmission, modification of neurotransmitter actions in specific brain nuclei remains challenging. Herein, we report a novel approach for neurotransmission modification *in vivo *by transplantation of stem cells engineered to take up the neurotransmitter dopamine (DA) efficiently through the action of the human dopamine transporter (hDAT). As a functional test in mice, we used voluntary alcohol consumption, which is known to release DA in nucleus accumbens (NAC), an event hypothesized to help maintain drug-seeking behavior. We reasoned that reducing extracellular DA levels, by engrafting into NAC DA-sequestering stem cells expressing hDAT, would alter alcohol intake.

**Methods:**

We have generated a neural stem cell line stably expressing the hDAT. Uptake kinetics of DA were determined to select a clone for transplantation. These genetically modified stem cells (or cells transfected with a construct lacking the hDAT sequence) were transplanted bilaterally into the NAC of wild-type mice trained to consume 10% alcohol in a two-bottle free-choice test for alcohol consumption. Alcohol intake was then ascertained for 1 week after transplantation, and brain sections through the NAC were examined for surviving grafted cells.

**Results:**

Modified stem cells expressed hDAT and uptaken DA selectively via hDAT. Mice accustomed to drinking 10% ethanol by free choice reduced their alcohol consumption after being transplanted with hDAT-expressing stem cells. By contrast, control stem cells lacked that effect. Histologic examination revealed surviving stem cells in the NAC of all engrafted brains.

**Conclusions:**

Our findings represent proof of principle suggesting that genetically engineered stem cells can be useful for exploring the role of neurotransmitters (or other signaling molecules) in alcohol consumption and potentially in other aspects of brain function.

## Introduction

It has been 50 years since Olds and Milner [1] described the existence of reward pathways in the brain, based on their experiments showing that electrical stimulation of certain brain areas is rewarding to rats. Today's understanding of common reward pathways in the brain involves the mesocorticolimbic circuitry consisting of dopaminergic cell bodies in the ventral tegmental area (VTA) and their projections to terminal areas of the prefrontal cortex and the "extended amygdala" (the NAC, substantia innominata, bed nucleus of the stria terminalis and amygdala). Rewarding stimuli such as food, sex, and drugs of abuse, including ethanol, result in the release of DA in terminal areas, particularly the NAC [2]. Although the dopaminergic mesocorticolimbic pathway is clearly involved in reward mechanisms, questions about the precise role of DA in drug addiction remain. We hypothesize that because the DAT regulates the concentration and duration of synaptic DA available to stimulate postsynaptic D_1 _and D_2 _receptors [3], overexpression of DAT should decrease the accumulation of released DA and reduce the ethanol consumption observed in mice.

To this end, we generated a cell line of C17.2 neural stem cells that stably overexpresses the hDAT and then transplanted these cells into the NAC of alcohol-preferring female C57BL/6J mice. Transplantation of embryonic neurons or neural stem cells into brains of animals serving as models of neural disorders has recently attracted more attention. For example, several studies have shown that transplantation of C17.2 cells into the CNS can repair a genetic defect such as dysmyelination [4] and that when overexpressing glucuronidase corrects lysosomal storage deficiency [5]. Ours is the first report of using stem cells for modification of neurotransmission in a model of drug preference. The plasticity and ease of genetic manipulation of these cells makes them ideal candidates for neurotransplantation designed to alter endogenous levels of a single molecule; in this case, the hDAT. By manipulating the expression of the hDAT, we sought selectively to affect dopaminergic neurotransmission and ethanol consumption.

## Materials and methods

### Animals and cell culture

Animal protocols and use were in strict accordance with the NIH *Guide for the Care and Use of Laboratory Animals *and were approved by the Institutional Animal Care and Use Committee at the University of Colorado Denver. Male Sprague-Dawley rats (*n *= 3; 175 to 225 g; Charles River Laboratories) housed on a 12-h light/dark cycle with *ad libitum *food and water were used in experiments examining synaptosomal DA uptake. We used female C57BL/6J mice from Jackson Laboratories (6 weeks old at the beginning of the study; 15 to 18 g) for studies of ethanol preference (*n *= 30) and chronoamperometry (*n *= 5). Mice were housed individually on a 12-h light/dark cycle with food and water *ad libitum*.

The C17.2 neural stem cells were derived from postnatal mouse cerebellum, express β-galactosidase [6], and graft well into rodent CNS. After transplantation, C17.2 cells differentiate into several types of neurons, astrocytes, and oligodendrocytes naturally occurring at the site of transplantation [7], and home into areas of brain damage [8]. These studies also demonstrate that C17.2 cells do not form tumors, as their growth in contact is inhibited. The C17.2 neural stem cell line and its clones were grown in DMEM/10% fetal bovine serum/5% horse serum, supplemented with glutamine (2 m*M*), sodium pyruvate (1 m*M*), and penicillin/streptomycin/Fungizone (100× containing 10,000 units penicillin, 10,000 μg streptomycin, and 25 μg amphotericin B; Gibco, Rockville, MD), in a 37°C incubator at 5% CO_2_.

### Plasmid construction and transfection of neural stem cells

To ensure a strong expression of hDAT in C17.2 cells after transplantation and differentiation, a combination of the cytomegalovirus enhancer and the chicken beta actin promoter in a pCAIP_2 _plasmid was used (kindly provided by Ernest Arenas of Karolinska Institutet). The construct contains the coding sequence of hDAT, an intron (IVS), an internal ribosome entry site (IRES), and the coding sequence of the puromycin resistance gene. The arrangement facilitates screening of stable transfectants that express hDAT. The hDAT cDNA was obtained from Jonathan A. Javitch, Columbia University. Exponentially growing C17.2 cells were trypsinized, plated into 10-cm tissue-culture dishes, and grown overnight in growth medium. Plasmid DNA (20 μg) was mixed with 0.5 ml of CaCl_2 _(0.25 *M*) and 0.5 ml BES-buffered saline (BBS), and incubated for 20 min at room temperature. DNA/CaCl_2_/BBS mixture was added drop-wise to the dishes, which were incubated overnight. C17.mock clones were transfected with plasmid not containing hDAT cDNA. The next morning, the cultures were rinsed twice with Hank's Balanced Salt Solution (HBSS) and returned to the growth medium. Forty-eight hours later, the medium was replaced with fresh medium containing puromycin (2.5 μg/ml). The medium containing puromycin was replaced daily. Six days after selection began, individual colonies of cells were gently lifted and transferred to individual wells of a 96-well plate. Clones were expanded when they became confluent. C17.hDAT and C17.mock clones were frozen after DA uptake was determined, within five to six passages after selection.

### Preparation of synaptosomes from dorsal striatum

After decapitation, rat brains were rapidly removed and placed in an ice-cooled dish where both hemispheres of dorsal striatum were dissected. Striatal tissue was homogenized in 2 ml of ice-cold phosphate buffer (3.3 m*M *NaH_2_PO_4 _+ 12.7 m*M *Na_2_HPO_4_) containing 0.32 *M *sucrose (pH 7.4) with a glass homogenization tube and a Teflon pestle. Homogenization was followed by centrifugation at 1,000 *g *for 12 minutes at 4°C. The supernatant was centrifuged at 12,500 *g *to isolate the P2 pellet and then resuspended at 15 mg/ml (wet weight of tissue). The resuspended P2 synaptosomal pellet was further washed by centrifugation to isolate the P4 synaptosomal pellet, which, on resuspension, was used in the uptake assay.

### [^3^H]DA uptake in synaptosomes

Chemicals were purchased from RBI/Sigma (St. Louis, MO, USA) with the following exceptions: (-)-cocaine HCl was obtained from the National Institute on Drug Abuse (Research Triangle Institute International, Research Triangle Park, NC), and [^3^H]DA was purchased from PerkinElmer Life Sciences (Boston, MA, USA). Striatal synaptosomal tissue was incubated with assay buffer (134 m*M *NaCl, 240 m*M *KCl, 65 m*M *CaCl_2_, 70 m*M *MgSO_4_, 3.3 m*M *NaH_2_PO_4_, 12.7 m*M *Na_2_HPO_4_, 11 m*M *glucose, and 1 m*M *ascorbic acid, pH 7.4) containing 1 μ*M *pargyline for 10 minutes at 37°C. [^3^H]DA was added at a final concentration of 0.5 n*M *(54.1 to 59.3 Ci/mmol) in addition to increasing concentrations of unlabeled DA (0.5, 5, 50, 500, 1,000, and 10,000 n*M*). The uptake assay was performed at 37°C for 3 minutes. Nonspecific uptake of [^3^H]DA was determined in samples incubated with 1 m*M *cocaine. The assay was halted by placing the samples on ice and adding 3 ml of ice-cold 0.32 *M *sucrose solution before filtering through Whatman GF/C glass microfiber filters with a cell harvester. The filters were washed an additional 2 times with the sucrose solution before being placed in scintillation vials filled with 4 ml of scintillation cocktail. Radioactivity was determined with liquid scintillation spectrometry. The protein concentration of the P4 synaptosomal pellet was determined according to the method of Bradford (1976), by using bovine serum albumin as the standard. K_m _and V_max _values for [^3^H]DA uptake were calculated with nonlinear regression fitting by using Graph Pad Prism software (San Diego, CA). All uptake studies involved three independent experiments with samples tested in triplicates.

### [^3^H]DA uptake in cultured neural stem cells

After thawing, clones were split once by trypsinization and plated into 12-well tissue-culture plates that were precoated with poly-L-lysine (Sigma; 10 μg/ml). Cells were grown for 48 hours before uptake was measured. The cells were rinsed and then assayed at 37°C in Krebs-Ringer HEPES buffer (KRH; 120 m*M *NaCl, 4.7 m*M *KCl, 2.2 m*M *CaCl_2_, 1.2 m*M *Mg SO_4_, 1.2 m*M *KH_2_PO_4_, 10 m*M *glucose, 10 m*M *HEPES, pH 7.4) supplemented with 10 μ*M *pargyline, 10 μ*M *ascorbic acid, and 10 μ*M *catechol. Assays (1 ml) included 50 n*M *[^3^H]DA (54.7 to 59.3 Ci/mmol). Nonspecific [^3^H]DA accumulation was determined in the presence of 1 m*M *(-)cocaine hydrochloride. After 10 minutes of incubation at 37°C, uptake was terminated by quickly washing the cells 3 times with 1 ml of ice-cold KRH. Cells were then solubilized in 0.5 ml of 3% trichloroacetic acid for 60 minutes with gentle shaking. Accumulated [^3^H]DA was determined by liquid scintillation counting. Clones showing robust uptake at 50 n*M *[^3^H]DA were then chosen for further studies of saturation kinetics in the presence of various concentrations of DA, or selectivity in the presence of 1 μ*M *GBR 12909, desipramine, or fluoxetine. Kinetic parameters were calculated as described earlier.

### Stem cell transplantation

C17.hDAT or C17.mock transfected cells were thawed into a 60-mm tissue-culture plate 4 to 7 days before transplantation. They were rinsed 4 to 6 hours after thawing and then split by trypsinization after 3 or 4 days, when they were 85% confluent. On the day of surgery, cells were used only if less than 90% confluent. They were trypsinized, spun at 300 *g*, and rinsed 4 times with HBSS before resuspension in HBSS at a density of 50,000 cells/μl. The cells were kept above ice until transplanted. Cells left over after surgery were plated into DMEM growth media and allowed to attach before staining with x-gal to verify β-galactosidase expression. Mice were anesthetized with isoflurane, and holes were drilled in the skull bilaterally at 1.4 mm anterior, and ±0.8 mm lateral to bregma (coordinates from [9]). A stainless steel guide cannula (27 gauge) with solid blunt insert was lowered 3 mm below the dura. The solid insert was then replaced with a 29-gauge cannula, which extended 1.5 mm past the end of the guide cannula and was attached to PE tubing and a 10-μl Hamilton syringe. Then 100,000 cells were injected from the cannula at a rate of 1 μl/min, while the cannula was raised 1 mm, depositing the cells over a 1-mm track. The cannula was left in place for an additional 2 minutes before removing it from the brain. Three groups of mice (nine per group) received either bilateral saline injection into the NAC, or bilateral transplants of C17.hDAT cells, a clone with functional DA transporter, or C17.mock, a clone mock-transfected with an empty plasmid.

### Two-bottle choice test

Alcohol preference was tested in 30 mice by introducing two water bottles for 3 days, and then replacing the water in one of the bottles with ethanol (3% wt/vol for 6 days, then 10% wt/vol for 8 days). Body weight and the volume of water and ethanol consumed from each bottle were measured every 3 days, at which time fresh ethanol and water were provided, and the positions of the bottles switched. The mice were given 3 days without ethanol before transplantation, followed by 3 days of recovery before preference was again tested with 10% ethanol in one of the two bottles. The ratio of the volume of ethanol to the total fluid consumed was calculated. Ethanol consumption was calculated in grams ethanol/kg body weight/day. Three mice were eliminated based on their pretransplantation ratios (<0.55), leaving nine mice per group for transplantation (27 mice total). Posttransplant alcohol consumption was not measured for one animal in the C17.mock group because it died postsurgically. All animals that survived transplantation had surviving bilateral grafts, and all of these animals were included in the alcohol-consumption analysis.

### Electrochemical recording of DA clearance

The method used is explained in detail in Hoffman *et al*. [10] and modified by Gulley *et al*. [11]. DA clearance was determined in C17.mock clone and C17.hDAT clone 5 to 7 days after transplantation into the right and left, respectively, cerebral cortex of each of the five mice used. Mice were killed by cervical dislocation, and their brains were rapidly chilled in ice-cold artificial cerebrospinal fluid (aCSF; 126 m*M *NaCl, 2.9 m*M *KCl, 1.5 m*M *MgCl_2_, 2.5 m*M *CaCl_2_, 1.4 m*M *NaH_2_PO_4_, 10 m*M *glucose, 25 m*M *NaHCO_3 _and 200 μ*M *ascorbate, continuously bubbled with 95% O_2_/5% CO_2_; pH 7.4). Coronal brain sections (400 μm) containing the transplanted cell tracks in the cerebral cortex were cut with a Vibratome (Ted Pella, Inc., Redding, CA). After slice recovery at 22°C in aCSF for ≥1 hour, slices were transferred to a recording chamber, perfused with aCSF at a rate of 2 ml/min, and maintained at 32° to 33°C. High-speed chronoamperometric measurements were carried out by using an IVEC-10 system (Medical Systems, Greenvale, NY). Single-carbon-fiber electrodes (30 μm diameter, coated with 5% wt/vol Nafion solution; Sigma-Aldrich, St. Louis, MO) were calibrated *in vitro *and used only if their responses were linear to DA (*r*^2 ^≥ 0.997) and selective (≥1,000:1) for DA over ascorbic acid. Electrode-micropipette assemblies were constructed by attaching a micropipette (A-M Systems, Everett, WA; 10- to 15-μm tip diameter) to the electrode at a distance of 200 to 250 μm. This assembly was lowered to a depth of 100 to 150 μm from the slice surface into the cerebral cortex just adjacent to the surgical track left from the cell implantation. To detect DA, square-wave pulses of 0.00 to +0.55 V, with respect to an Ag/AgCl reference electrode, were applied at a frequency of 5 Hz. DA (200 μ*M *in phosphate-buffered saline (pH 7.4, adjusted with NaOH) and 100 μ*M *ascorbic acid) was pressure-ejected (6 to 15 psi for 0.1 to 2 seconds; Picospritzer II; General Valve Corp., Fairfield, NJ) until a maximal DA signal amplitude (A_max_) of 1 to 3 μ*M *was obtained. DA was pressure-ejected with these settings kept constant at 5-minute intervals for a minimum of three consistent recordings (≤15% variation in A_max _values) at a particular recording site. Clearance time (T_80_; seconds), the time for the signal to increase to A_max _and then to decay by 80%, and clearance rate (μ*M*/sec), the change in DA signal amplitude between the T_20-60 _time points and the most linear portion of the DA signal-decay curve, were measured. For each transplant, two to four recording sites in different brain slices were sampled, and these were used to calculate mean values for each parameter for the two types of stem cells. Mean values ± SEM are reported for *n *= number of mice.

### Immunohistochemical analysis

Animals were deeply anesthetized and transcardially perfused with saline followed by 4% paraformaldehyde. The brains were cryoprotected in 30% sucrose for 2 days before sectioning at 40 μm. Sections were rinsed and quenched in 1% H_2_O_2 _for 10 minutes before blocking in 10% normal goat serum in Tris-buffered saline containing 1% BSA and 0.1% Triton X-100 (TBST). Sections were incubated in a primary antibody to hDAT (1:200, Chemicon) overnight at room temperature. After rinsing, sections were incubated in anti-rat IgG (1:250, Vector) for 1 hour at room temperature. After additional rinses, incubation with ABC solution (Vectastain, Vector) for 1 hour and then nickel-enhanced diaminobenzidine (Vector) permitted visualization of labeled cells.

### Beta-galactosidase activity assay and localization of transplanted cells

Cells in culture or sections were stained for β-galactosidase by incubating with 5-bromo-4-chloro-3-indolyl-β-D-galactosidase (X-gal) (1 mg/ml in 5 m*M *potassium ferrocyanide/potassium ferricyanide and 2 m*M *MgCl_2 _buffer) for 2 to 18 hours at 37°C. Because the actual number of transplanted cells would be impossible to count, the localization of transplanted cells in each group was summarized by scoring the relative abundance of β-galactosidase-positive cells present in a grid location overlaid on three stained sections from each mouse. Sections showing the most cells bilaterally were chosen for analysis, roughly approximating rostral, middle, and caudal extent of the track. Some transplants were localized bilaterally in more anterior sections of the NAC, and some were slightly more caudal in the NAC. Each square of the grid was scored as containing no cells (0), a few sparse cells in less than one fourth of the square (1), sparse cells covering about half of the square (2), densely packed cells occupying about half of the square (3), densely packed cells occupying three fourths of the square (4), or densely packed cells entirely filling the square (5). The scores of each animal in a group of either anterior or posterior NAC were summed and used as the z coordinate for a colorized xyz isocontour plot (SigmaPlot; SysStat, Point Richmond, CA), where x and y represented the square coordinates of the grid (0, white; 1, purple/blue; 2, dark green; 3, light green; 4, yellow; 5, orange/red). For clarity, details of this method are illustrated in example micrographs in Supplementary figure S1 in Additional file 1. The number of sections contributing to each cumulative plot was between 9 and 15.

### Statistical analysis

Data are reported as the mean ± SEM. One mouse from each group was eliminated because their alcohol preference ratio was <0.55 before transplantation. The inhibition of DA uptake by selective inhibitors was analyzed by one-way ANOVA by using SigmaStat with Tukey *post hoc *comparisons. Body weight and total daily fluid consumption were averaged to yield pre- and postsurgery values. Alcohol consumption (grams ethanol/kg body weight/day) was averaged at each concentration of ethanol before transplant and after transplant. Ethanol consumption, total fluid consumption, and body-weight data were analyzed by two-way ANOVA by using version 6.5 of GB-STAT (Dynamic Microsystems Inc., Silver Spring, MD) with DAY as a repeated measure. *Post hoc *analyses were done with the Fisher LSD test.

## Results

### Generation and characterization of hDAT-expressing neural stem cells

**Figure 1:**
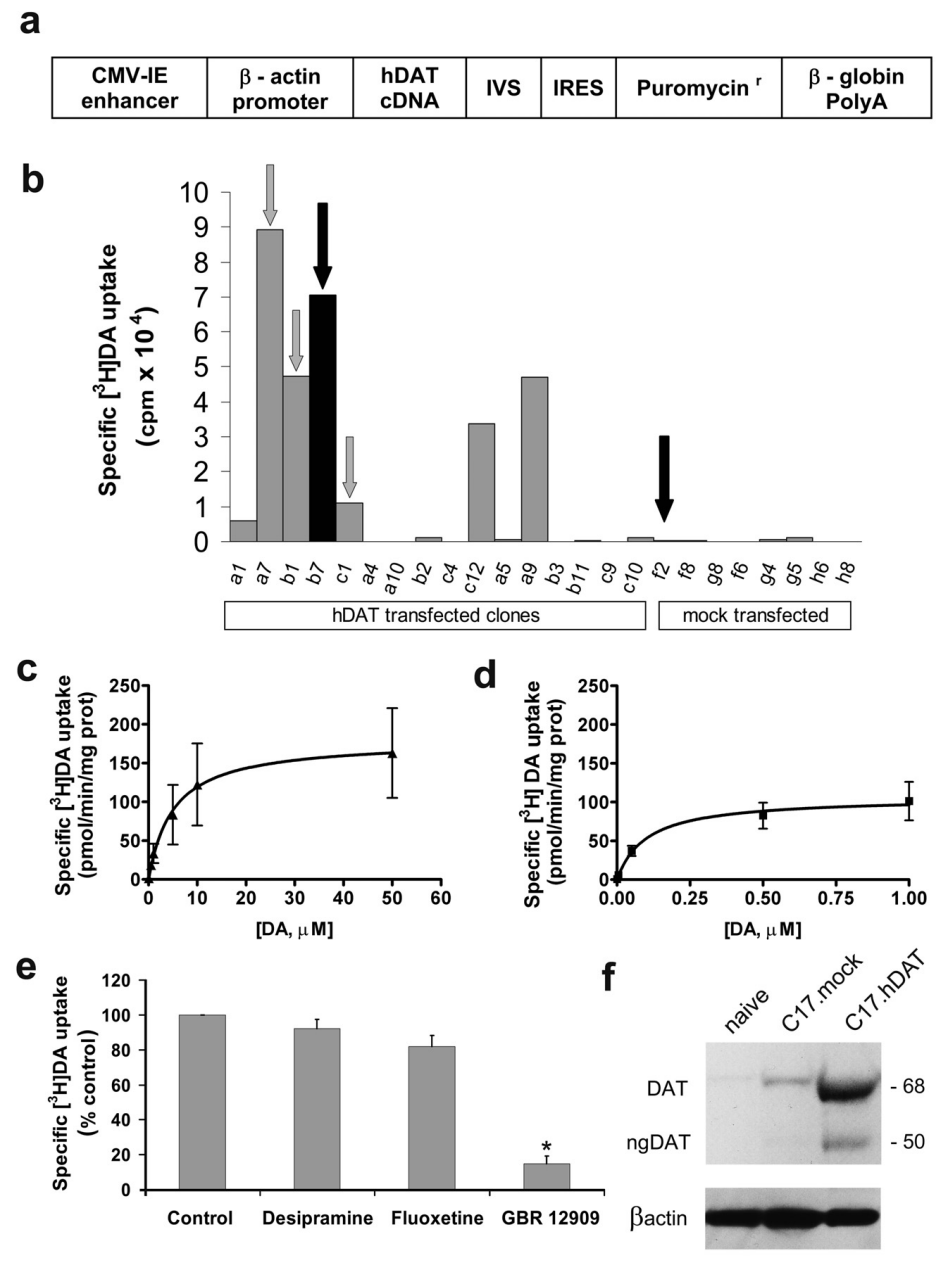
**Human DAT construct used in the stable transfection of C17.2 neural stem cells and characterization of its functional expression in selected clones**. **(a) **Expression vector used for transfection. The construct contained the cytomegalovirus (CMV) enhancer element, chicken β-actin promoter, the coding sequence of hDAT, an intron (IVS), an internal ribosome entry site (IRES), and the coding sequence of the puromycin resistance gene. **(b) **Clones that survived selection with puromycin were screened for specific [^3^H]DA uptake (±1 m*M *cocaine). Several clones were chosen for expansion and further analysis based on their uptake of DA (arrows). Clones b7 (C17.hDAT) and f2 (C17.mock), identified by black arrows, were chosen for the transplantation and alcohol-consumption experiments. **(c) **Kinetic analysis of specific [^3^H]DA uptake in a C17.hDAT clone. Three independent experiments revealed average values for *K*_m _of 5 ± 1 μ*M *and *V*_max _of 180 ± 5 pmol/min/mg protein. **(d) **In similar [^3^H]DA-uptake kinetic experiments, rat striatal synaptosomes had values for *K*_m _of 0.10 ± 0.02 μ*M *and *V*_max _of 108 ± 27 pmol/min/mg protein (*n *= 3). **(e) **Selectivity experiments in the C17.hDAT clone revealed that [^3^H]DA uptake was blocked only by the DAT inhibitor (GBR 12909, 1 μ*M*), but not by selective inhibitors of norepinephrine or serotonin transporters, desipramine, or fluoxetine (1 μ*M*), respectively. *P < 0.01 compared with control, Tukey test (three independent experiments). **(f) **Western immunoblot for hDAT showing both mature, glycosylated (DAT) and immature, nonglycosylated forms of hDAT (ngDAT) in the C17.hDAT clone. Beta-actin was detected as a control for protein loading.

Because reliable and enduring expression of functional hDAT is required for transplantation, we first generated clones of C17.2 neural stem cells that stably expressed hDAT under the control of a chicken β-actin promoter and a cytomegalovirus enhancer. This promoter has been used successfully to express a transcription factor, Nurr1, and glial cell line-derived neurotrophic factor in C17.2 stem cells [12,13]. Two plasmids were created, one with the hDAT cDNA (Figure 1a) and the other (mock plasmid) lacking any cDNA at the position of hDAT cDNA. Cells were transfected with these plasmids, by using the calcium phosphate method, and were subsequently grown in the presence of puromycin for selection.

After selection with puromycin, 36 hDAT-transfected colonies and 36 mock plasmid colonies were grown individually in 96-well plates. Sixteen of the hDAT clones and eight of the mock plasmid clones were randomly chosen, and specific [^3^H]DA uptake was determined (50 n*M *[^3^H]DA ± 1 m*M *cocaine). Seven of 16 hDAT clones assayed exhibited moderate to high levels of uptake (Figure 1b). Specific [^3^H]DA uptake was undetectable in all mock plasmid transfected clones, as well as in nine of the hDAT clones. Four of the hDAT clones (a7, b1, b7, and c1) that exhibited a wide range of uptake levels were chosen for full kinetic uptake analyses. Results are shown for the b7 clone (Figure 1c), which was selected for transplantation experiments (see later), and from this point on, referred to as C17.hDAT. The clones exhibited affinities (*K*_m_) between 5 and 9 μ*M *and a range of saturation velocities (*V*_max_) between 43 and 180 pmol/min/mg protein. To achieve efficient uptake kinetics from genetically modified clones, their uptake should resemble that of the naturally occurring DAT. The *V*_max _values from our DAT clones examined were found to be comparable to those obtained from rat striatal synaptosomes, with somewhat lower affinities (*K*_m _of 0.10 ± 0.02 μ*M *and *V*_max _of 108 ± 27 pmol/min/mg protein; Figure 1d) and those recently reported for rat NAC synaptosomes (*K*_m _of 0.20 ± 0.04 μ*M *and *V*_max _of 57 ± 11 pmol/min/mg protein) [14]. The four clones also exhibited selectivity for [^3^H]DA transport, as GBR 12909 (DAT inhibitor) blocked specific [^3^H]DA uptake by 90% (Figure 1e). By contrast, desipramine (norepinephrine transporter inhibitor) and fluoxetine (serotonin transporter inhibitor) had no effect on the uptake.

In addition to the efficient and selective uptake of DA resembling naturally occurring DAT, other reasons for choosing the C17.hDAT clone for transplantation included (1) a consistent β-galactosidase expression in more than 95% of this clone's cells, an asset for identification of the cells in transplants; (2) a good proliferative capacity crucial for expansion before transplantation; and (3) a high survival rate after transplantation into the CNS. Although the presence of functional DAT is an unquestionable indication of the transporter's appropriate insertion into the cell membrane, we additionally quantitated the expression levels of hDAT by using Western immunoblotting (Figure 1f). This analysis revealed that the C17.hDAT clone expressed two forms of hDAT. The first was a 68-kDa mature form, which is glycosylated and exclusively found inserted into the cell membrane [15]. The second form of hDAT was a band at 50 kDa, consistent with an immature, unglycosylated form usually found in the cytoplasmic compartment. The C17.hDAT clone expressed about 95% of the hDAT as the mature (68 kDa) form and about 5% as the immature (50 kDa) form. The naïve C17.2 stem cells and the mock-transfected f2 clone (C17.mock) contained only trace amounts of immunoreactivity for hDAT.

### Establishing alcohol-drinking model in wild-type mice and transplantation of hDAT-expressing neural stem cells into the NAC

**Figure 2:**
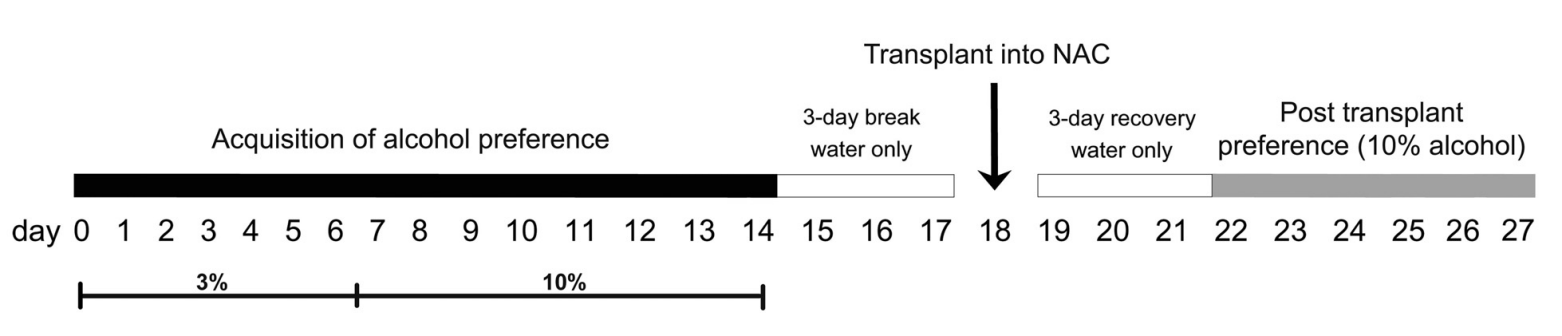
**Experimental timeline**. Animals were allowed free choice between water or ethanol (3% for days 1 to 6, 10% for days 7 to 14). Transplants (saline control, C17.mock, or C17.hDAT; *n *= 9 in each group) into NAC, were performed after 3 days off alcohol, and then preference for 10% ethanol was measured again after 3 days of recovery from surgery for an additional 6 days. All animals were sacrificed 10 days after transplantation.

**Figure 3:**
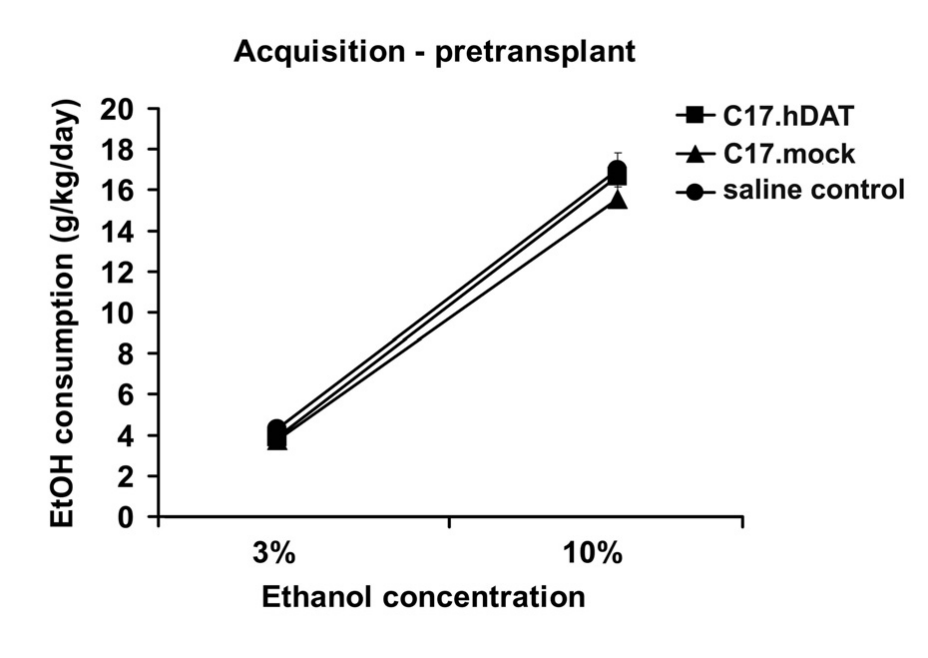
**Determination of alcohol consumption before transplantation**. Ethanol consumption (g/kg/day) was measured during the acquisition of the alcohol-preference phase over 14 days before transplant (day 17) in each group of animals. Consumption increased with time and ethanol concentration.

Before transplantation into the NAC, the mice were allowed to develop a preference for alcohol drinking, the variable that we would then attempt to alter by grafting hDAT-expressing stem cells. A timeline for the *in vivo *experiments is shown in Figure 2. Singly housed mice were given free and unrestricted choice of water or ethanol from two bottles for 24 hours a day in their home cage (two-bottle preference test). The initial ethanol concentration was 3% for 6 days and then increased to 10% for the next 8 days. By the end of this training period and before transplantation, mice consumed on average 16 g/kg/day of alcohol, and about 75% of their total fluid intake was from the alcohol-containing bottle. As illustrated in Figure 3, alcohol consumption increased similarly in all mice before transplantation. Initially the preference ratio (proportion of ethanol relative to total fluid consumed) was around 0.50, but gradually increased until day 14, when most mice had ratios above 0.70 (mean, 0.75 ± 0.04; *n *= 30). All animals with preference ratios above 0.55 (*n *= 27) were included and divided equally based on their preference ratio among the three groups receiving transplants. For 3 days before, as well as 3 days after, transplantation, all mice were given only water to drink to circumvent any potential adverse effects of ethanol during surgery and recovery.

On day 18, two groups of animals were transplanted bilaterally with 1 × 10^5 ^viable cells (either C17.hDAT clone or C17.mock clone) in the NAC on each side of the brain. Saline injected bilaterally into the NAC served as a surgical control (the third group that we refer to as saline control). Each transplant was delivered in a volume of 2 μl, by using a stainless steel transplant cannula (see Materials and methods). To assure that transplantation did not alter normal patterns of body weight and total fluid consumption, both parameters were measured every 3 days throughout the study. Body weight increased in all groups over the course of the experiment (*F*_1, 23 _= 72.35; *P *< 0.0001; Table 1). However, it did not differ among the transplant groups (*F*_3, 23 _= 0.77; *P *= 0.47), and no significant interaction was found (*F*_2, 23 _= 1.24; *P *= 0.31). The values are similar to body weights reported for female C57BL/6J mice between 6 weeks and 10 weeks of age (Jackson Laboratories). Similarly, total fluid consumption increased over the course of the experiment (*F*_1, 24 _= 9.27; *P *= 0.006; Table 1). However, it did not differ among groups (*F*_3, 24 _= 0.19; *P *= 0.83), and no significant interaction was observed (*F*_2, 24 _= 1.16; *P *= 0.33). The lack of differences in body weight and fluid consumption between the groups indicates that the transplantation surgery was not detrimental to the general health of the animals. Additionally, no noticeable changes were observed in the behavior of the mice after surgery.

**Table 1:** Body weight and daily fluid consumption before and after transplantation (TX)

	Body weight (g)		Average daily fluid consumption (ml/day)	
		
	Before TX	After TX	Before TX	After TX
Saline	18.8 ± 0.36	19.8 ± 0.36	5.4 ± 0.21	5.7 ± 0.26
C17.mock	18.2 ± 0.29	19.3 ± 0.54	5.0 ± 0.14	5.7 ± 0.24
C17.hDAT	18.5 ± 0.26	20.0 ± 0.36	5.2 ± 0.21	5.6 ± 0.33

C17.hDAT, C17.2 neural stem cell line stably expressing a construct containing the hDAT sequence; C17.mock, C17.2 neural stem cell line stably expressing a construct lacking the hDAT sequence; TX, transplantation.

### Characterization of hDAT-expressing neural stem cell grafts

**Figure 4:**
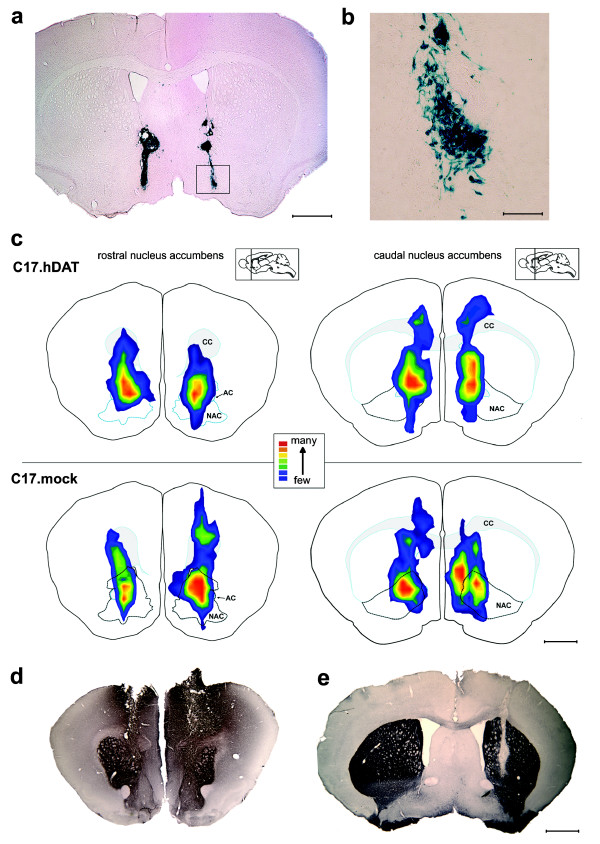
**Histologic analysis of grafted neural stem cells**. **(a) **A representative coronal section from a mouse transplanted with C17.hDAT clone. Section stained for β-galactosidase by using x-gal histochemistry revealed bilateral distribution of surviving grafted cells 10 days after transplantation. **(b) **Magnified area of the graft shows cells with various morphologies; some extending processes. **(c) **Summary of the localization of transplanted cells. Relative abundance of x-gal-positive cells in all mice in both groups receiving cell transplants is represented as a colorized xyz isocontour plot (for details, refer to Materials and methods). Cumulative scores from three sections per mouse ranged from few cells (1, blue) to many densely packed cells (5, red). For clarity, details of this method are illustrated in example micrographs in Supplementary Figure S2 in Additional file 3. **(d) **Coronal section at the level of NAC stained with an antibody against DAT. Although the antibodies used do not distinguish endogenous DAT from that found in the transplanted C17.hDAT cells, the ectopic expression of DAT evident in the cortical region of the transplant track is consistent with the exogenous hDAT. Such ectopic placement of a transplant is not representative of other animals examined in this study. **(e) **In contrast, a transplant of the C17.mock stem cells into striatum, shown here in a coronal section, is not labeled with the anti-DAT antibody and is clearly visible as a vertical clear track in the right hemisphere, which labeled strongly, revealing the endogenous DAT. CC, corpus callosum; AC, anterior commissure. Scale bars = 1 mm for panels a and c through e; and 200 μm for panel b.

Graft placement, determined from X-gal staining (Figure 4a and 4b) 10 days after transplantation, revealed that the majority of transplanted cells remained either within the transplant track or in its immediate vicinity, with a few cells seen 100 to 200 μm away from the track. X-gal staining was evident in all of the mice transplanted with C17.hDAT or C17.mock stem cells. The localization of the transplanted cells in all of the mice in each of these two groups was summarized by scoring the abundance of transplanted cells in three representative coronal sections from each mouse (Figure 4c). The transplants were targeted into the NAC, which is a relatively small structure in the mouse brain, reaching a maximal width of 2 mm [9]. Although current technology makes it difficult to achieve consistently more precise graft placement, continual refinements in the transplantation technique and equipment will enable more precise targeting in the future.

Currently available anti-DAT antibodies used recognize both endogenous (murine) and exogenous (human) DAT. Nonetheless, we were able to observe (albeit rarely) ectopic localization and elevated levels of DAT immunoreactivity in sections obtained from animals receiving the C17.hDAT clone (Figure 4d), whereas in contrast, transplants of the C17.mock neural stem cells completely lacked DAT-immunoreactivity (Figure 4e).

To confirm that C17.hDAT cells retain their ability to take up DA after transplantation, we measured the rate of DA clearance in transplanted cells in a group of five mice. Clearance of locally applied exogenous DA, as measured by high-speed chronoamperometric recording, is determined to a large extent by the activity of the DAT [11,16]. Cortex was used as the site for transplantation because its relatively sparse DA innervation and low DA clearance rate, relative to striatum, provides a low back-ground clearance and would thereby enhance detection of any increase in clearance rate due to the hDAT-expressing stem cells. Consistent with this idea, the DA clearance rate in brain slices from naïve rats is threefold higher in striatum (0.115 ± 0.035 μ*M*/sec; *n *= 4) than cerebral cortex (0.036 ± 0.009; unpublished observations, Gaynor A. Larson and Nancy R. Zahniser). Similarly, we observed a fourfold higher striatal clearance rate in brain slices from naïve mice (striatum, 0.148 μ*M*/sec; cortex, 0.032; *n *= 2).

Here, we used this method to compare DA clearance in C17.hDAT and C17.mock grafts transplanted 5 to 7 days earlier into mouse cerebral cortex. Brain slices were prepared from five mice, each of which had the C17.mock control cells implanted into the right cerebral cortex, and the C17.hDAT cells, into the left cortex. A trend for a faster clearance rate (20% increase) was noted in the C17.hDAT transplants (0.054 ± 0.009 μ*M*/sec), compared with C17.mock (0.045 ± 0.006; Table S1 in Additional file 2 and Supplementary figure S2 in Additional file 3). Both clearance rates were somewhat faster than the rate we observed previously in naïve mouse cortical slices (see earlier).

### Transplantation of DA-sequestering neural stem cells reduces intake of alcohol in wild-type mice

**Figure 5:**
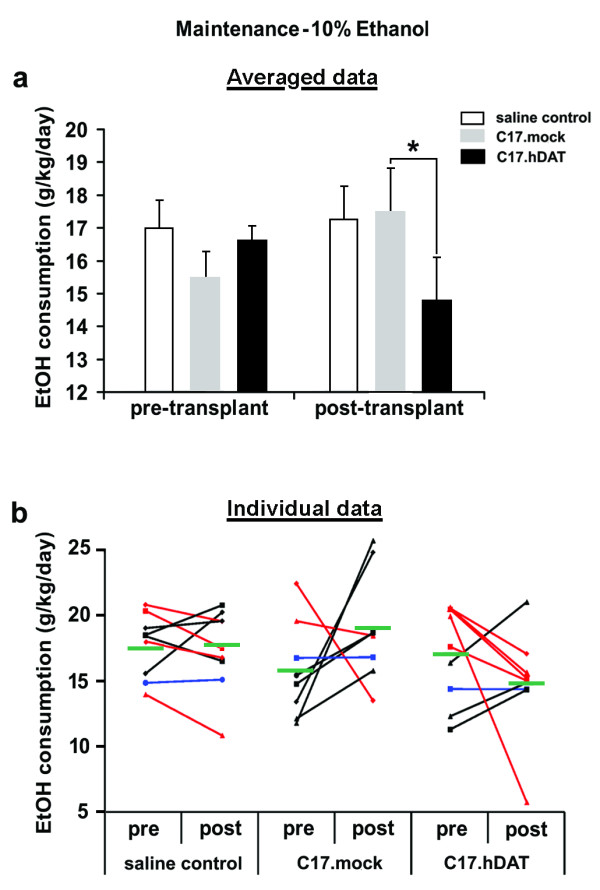
**Comparison of alcohol consumption before and after transplantation**. **(a) **Consumption of 10% ethanol was lower after transplantation in mice transplanted with C17.hDAT cells when compared with mice receiving C17.mock cells. While average pre- versus posttransplant consumption was reduced in most of the C17.hDAT animals, it remained unchanged or slightly elevated in saline control and C17.mock groups, respectively. *P < 0.05 comparing posttransplant C17.hDAT with posttransplant C17.mock group. **(b) **Average consumption of 10% alcohol before and after transplantation in individual animals. The effects on consumption between pre and post measurements are depicted by different line colors: red (reduction in consumption), black lines (increase), and blue (no change). Mean group value is represented by a green horizontal bar for each group. Although five of nine C17.hDAT animals reduced their consumption, only two of eight C17.mock animals did. Pre, before transplantation; post, after transplantation.

Measurement of alcohol consumption was resumed 3 days after the surgery for an additional 6 days. In the majority of the animals that received saline or C17.mock cells, the preference for 10% ethanol remained unchanged or slightly increased after transplantation (Figure 5). Analysis of the average alcohol consumption before transplantation (3% and 10%) and postsurgically showed a significant main effect of Day (*F*_2, 46 _= 190.85; *P *< 0.0001), but not Group (*F*_2, 23 _= 0.99; *P *= 0.39). Additional analysis revealed a significant interaction of Day and Transplant group (*F*_4, 46 _= 2.86; *P *= 0.034). After transplantation, the mice in the C17.hDAT group significantly reduced their ethanol consumption by 18% from 17 to 14 g/kg/day when compared with the C17.mock group (*P *< 0.05; Figure 5a). Values for individual animals in each group are shown in Figure 5b.

## Discussion

Here, we demonstrate that overexpression of DAT alters ethanol drinking in C57BL/6 mice. Because mesocorticolimbic DA is postulated to contribute to alcohol consumption, we transplanted neural stem cells modified to overexpress the hDAT into the NAC of C57BL/6 adult female mice. We postulated that grafted C17.hDAT cells that express high levels of the DAT should clear the extracellular DA faster than the surrounding tissue. High-speed chronoamperometric measurements showed a trend toward faster DA clearance rates in cortical C17.hDAT transplants compared with C17.mock transplants. The reduction in alcohol intake that was observed in mice with the C17.hDAT transplants in NAC is consistent with the idea that NAC DA might affect ethanol consumption.

Our ethanol-consumption data are in agreement with studies showing that D_1 _and D_2 _receptor antagonists, injected systemically or locally into the NAC, decrease alcohol consumption and operant responding to alcohol [17,18]. Mice lacking D_1 _receptors also show decreased ethanol consumption in a two-bottle choice test compared with wild-type mice [19]. Extracellular DA levels are increased in the NAC by systemic alcohol administration [20-23]. These findings are consistent with the theory that DA neurotransmission in the NAC is necessary for the rewarding properties of alcohol. However, despite numerous studies examining the involvement of the dopaminergic system in reward, the precise role for DA in drug addiction is still far from clear.

The DAT is the primary physiologic mechanism for terminating DA neurotransmission in the CNS. By inhibiting the DAT, drugs of abuse like cocaine and phencyclidine preferentially increase extracellular DA in NAC, as compared with dorsal striatum. Ethanol also elevates DA levels in the NAC. However, unlike cocaine, ethanol does this by increasing the firing of DA neurons in the VTA [24]. Alcohol-preferring rats will self-administer ethanol into the VTA [25]. Ethanol exerts its actions by potentiating GABAergic inhibition of inhibitory interneurons in the VTA [26,27], leading to disinhibition of DA neurons. As with other drugs of abuse, the increased firing of VTA neurons results in enhanced release of DA in the NAC [22,28,29].

Some studies have reported that D_1 _and D_2 _receptor agonists, as well as pharmacologic agents that elevate DA levels, also reduce ethanol self-administration [30-32]. Conceivably, the reduced drug-seeking behavior resulting from activation of D_1 _or D_2 _receptors in these studies may be due to either a decrease in the rewarding properties of alcohol or a feedback-mediated elevation of endogenous DA, which reduces the drive for alcohol [19]. Enhancing extracellular DA in the NAC with GBR12909, a DA-uptake inhibitor, decreased DA levels in the VTA, suggesting the presence of a negative-feedback loop in the regulation of DA release [33]. The regulation of DAT by alcohol might also have a role in the control of alcohol intake, as supported by the observation that chronic (8-week) consumption of 15% alcohol increases DA uptake in the NAC of selectively bred high-alcohol-drinking (HAD) female rats [34]. Although our study differs by using wild-type female mice in a shorter (27-day) experimental paradigm with a maximum alcohol concentration of 10%, both studies imply that plasticity in the reward circuitry involving DAT might be a modifier of alcohol drinking.

Recently, two alternative approaches for overexpression of DAT have been used. The first is a global one achieved by creating transgenic mice harboring a bacterial artificial chromosome containing the murine DAT gene [35]. Although these mice display an increased locomotor response to amphetamine compared to wild-type mice, they have a reduced operant response to a natural sweet-food reward, a finding that in principle supports our observations. A second study used direct injection into rat NAC of a lentiviral construct encoding rat DAT, which led to an impulsive and risk-prone phenotype [36]. This approach varies from ours in that lentivirus-infected neurons in the NAC can retrogradely transport DAT product into the VTA, whereas the hDAT expressed by C17.hDAT cells in our study is not transported and remains within the confines of the engrafted area.

Alcohol-preferring strains of rats, as well as the C57BL/6J mouse used here, have lower basal levels of dopaminergic function (decreased DA levels and turnover) than do non-alcohol-preferring strains [30,37,38]. In the C57BL/6J mice, enhancing synaptic DA decreased ethanol consumption, an effect that could be mimicked with DA-receptor agonists or blocked by DA-receptor antagonists [30]. These results suggest that voluntary ethanol consumption is higher in these mice because of increased drive (that is, they need more ethanol to get the same reward). If this were simply the case, we might have expected that the overexpression of DAT (lowering the amount of released DA to interact with postsynaptic receptors) would increase drive and ethanol consumption. Our finding of decreased ethanol consumption after the overexpression of DAT is inconsistent with the theory that increased preference and consumption in C57BL/6 mice is due to increased drive. The alternative theory, as to why the hypodopaminergic C57BL/6 mouse is alcohol preferring, is that the ethanol-induced release of DA on top of a low baseline is perceived as a larger reward (signal-to-noise difference). However, by overexpressing DAT, we might be diminishing the actions of DA and thereby reducing the stimulation of postsynaptic DA receptors, leading to decreased consumption. Thus, our mice are not simply hypodopaminergic, but might rather have dopaminergic neurotransmission through the NAC-reward pathway selectively inhibited.

Because we have measured DA clearance by stem cell transplants within cerebral cortical slices, in future experiments, both behavior and DA clearance must be measured simultaneously in the mice to assess whether a relation exists between changes in ethanol drinking and DA uptake by the C17.hDAT stem cells transplanted into the NAC. It is intriguing to speculate that stem cells engineered to express hDAT will adjust their capacity to clear DA, based on the transplant location in the brain or the level and duration of exposure to ethanol or both. Although the current study was not specifically designed to answer this question, addressing this issue represents an important direction for future explorations.

We chose to examine female C57BL/6J mice in our study based on the reported higher preference seen in females than males of this strain of mice. Because we did not use high and aversive concentrations of ethanol for our posttransplantation preference, an increase in consumption or preference ratio would have been detectable in our study. Lesions of the NAC with 6-hydroxydopamine decrease the acquisition, but not the maintenance of alcohol preference in alcohol-preferring rats [39]. This study suggests that preference, once established, is difficult to disrupt. It is possible that the observed effect on alcohol consumption would have been greater in magnitude if we had performed the transplants before the acquisition of alcohol preference. Alternatively, stem cells engineered to support higher dopamine clearance *in vivo *might also produce a larger reduction in ethanol consumption. The extent to which the posttransplantation decreases in alcohol intake might reflect changes in the reinforcing properties of ethanol should be confirmed in the future by using operant self-administration.

Studies presented here examine a very short experimental period. Future experiments will be designed to examine long-term survival of the transplanted stem cells, expression of hDAT, DA clearance, and to answer whether the effects on alcohol consumption persist. Long-term outcomes assessed over several weeks (or longer) will be therefore necessary to test our approach fully and to determine its broader usefulness.

Blood ethanol concentration (BEC) depends on alcohol intake and on the pharmacokinetics of alcohol metabolism, both of which vary between species and genders. Time of sampling is also critical for the estimation of BEC. In nocturnal animals such as mice, more than 90% of daily alcohol intake occurs during the dark [40]. When access to alcohol is continuous, C57BL/6J females drink as much as twice the amount of 10% alcohol typically consumed by the males [41]. In one report, male C57BL/6J mice drinking 10% ethanol in the unrestricted-access two-bottle preference test (same paradigm as the one used by us) exhibited BECs up to 150 mg/dl (in the dark) while consuming about 5 g/kg/day [40]. In a similar experiment, which used a drinking-in-the dark paradigm with a 4-hour 20% alcohol access, a mixed group of male and female C57BL/6J mice consumed on average 7 g/kg of alcohol in 4 hours, which resulted in a BEC of 100 mg/dl [42]. Because female mice used in our study had alcohol intakes between 15 and 18 g/kg/day, it is likely that the resulting BECs could have been even higher, particularly if measured in the dark. Because in humans, BECs around 80 mg/dl (the legal limit for driving while impaired in the United States) coincide with altered emotions, loss of control of fine motor movements, and affected driving performance [43], our consumption model is relevant to the human condition.

Our study demonstrates a novel strategy of using stem cell transplantation for moderating alcohol effects and intake, and our approach may provide future insights into better understanding of the mechanism of alcohol reward and addiction. This technique, when combined with the use of stem cells capable of metabolizing molecules of interest, could be of importance in studies requiring sequestration and degradation of molecular signals as well as neurotoxins. For example, glutamate neurotoxicity can be reduced by increasing expression of glutamate transporters [44]. Similar approaches could be used to study multidrug resistance-associated transporter proteins, which are important in the context of treatment of epilepsy and brain cancers [45] and transport across the blood-brain barrier for delivery of neuropharmaceuticals into the CNS [46].

## Conclusions

In summary, we determined that ethanol consumption is reduced by grafting stem cells overexpressing hDAT in the NAC. These data support the hypothesis that released DA in the NAC contributes to alcohol preference, and proposes DAT as a target for reducing alcohol drinking. Our findings also provide the first evidence for use of stem cells as vehicles for modification of neurotransmitter-mediated actions in the CNS.

## Abbreviations

AC: anterior commissure; BEC: blood ethanol concentration; CC: corpus callosum; CNS: central nervous system; DA: dopamine; DAT: dopamine transporter; HAD: high-alcohol drinking; hDAT: human DAT; IRES: internal ribosome entry site; IVS: intron; NAC: nucleus accumbens; VTA: ventral tegmental area; TX: transplantation.

## Competing interests

The authors declare that they have no competing interests.

## Authors' contributions

TNG, SMJ, MD, EYS, NRZ, BT, and WMZ contributed intellectually to the hypothesis and worked on the experimental design, interpretation of the data, and drafting of the manuscript. TNG, SMJ, BRH, MS, and GAL performed the experiments and collected data. All authors read and approved the final manuscript.

## Supplementary Material

Additional file 1 **Figure S1. Micrographs illustrating the method used for estimation of relative abundance of x-gal-positive cells in transplants**. The localizations of transplanted cells in the C17.hDAT group **(a) **and C17.mock. **(b) **were summarized by scoring the relative abundance of x-gal-positive cells present in a grid location overlaid on three stained sections from each mouse. Each square of the grid was scored and assigned a color in the isoplots in the following way: 0, containing no cells (white); 1, a few sparse cells in less than one fourth of the square (purple/blue); 2, sparse cells covering about half of the square (dark green); 3, densely packed cells occupying about half of the square (light green); 4, densely packed cells occupying three fourths of the square (yellow); 5, densely packed cells entirely filling the square (orange/red). Click here for file

Additional file 2 **Table S1**. Dopamine clearance rates *in vivo*. Click here for file

Additional file 3 **Figure S2. DA clearance parameters measured in C17.mock and C17.hDAT stem cells transplanted into mouse cerebral cortex**. Click here for file
