# Supplementary material for: Neurotransplantation of stem cells genetically modified to express human dopamine transporter reduces alcohol consumption
Source: Stem Cell Res Ther. 2010 Dec 1;1(5):36. doi: 10.1186/scrt36 (PMC3025438; doi:10.1186/scrt36)
Supplement: Additional file 1 — Figure S1. Micrographs illustrating the method used for estimation of relative abundance of x-gal-positive cells in transplants. The localizations of transplanted cells in the C17.hDAT group (a) and C17.mock. (b) were summarized by scoring the relative abundance of x-gal-positive cells present in a grid location overlaid on three stained sections from each mouse. Each square of the grid was scored and assigned a color in the isoplots in the following way: 0, containing no cells (white); 1, a few sparse cells in less than one fourth of the square (purple/blue); 2, sparse cells covering about half of the square (dark green); 3, densely packed cells occupying about half of the square (light green); 4, densely packed cells occupying three fourths of the square (yellow); 5, densely packed cells entirely filling the square (orange/red). [file scrt36-S1.PDF]

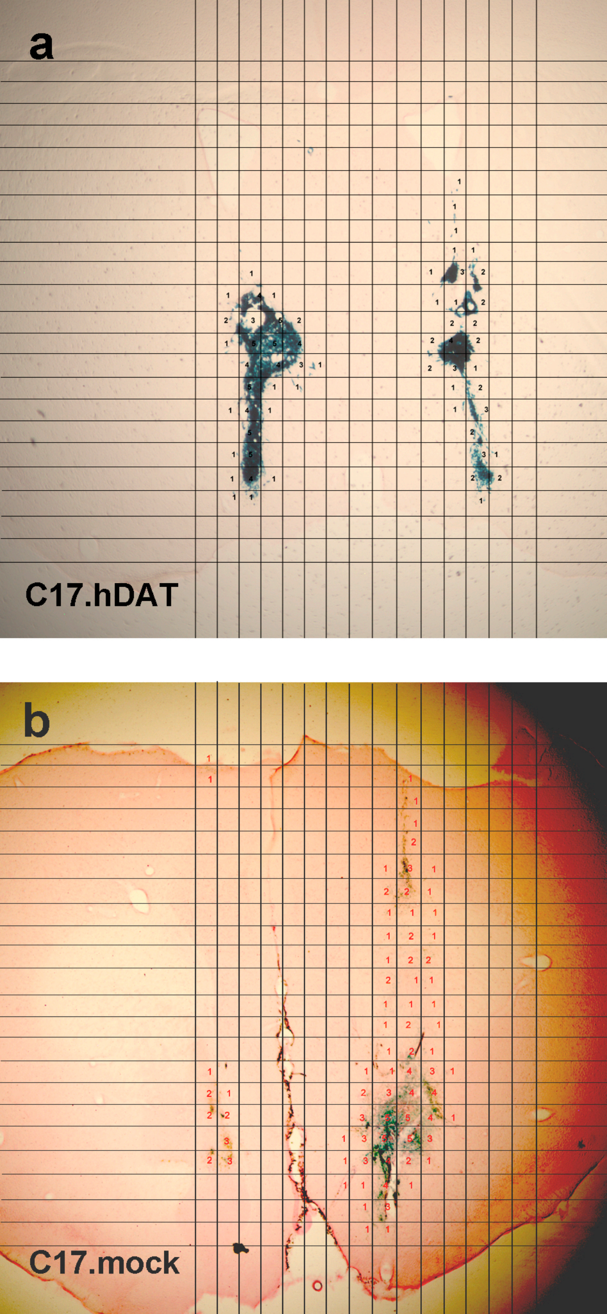
a
C17.hDAT
b
C17.mock
