# Supplementary material for: Neurotransplantation of stem cells genetically modified to express human dopamine transporter reduces alcohol consumption
Source: Stem Cell Res Ther. 2010 Dec 1;1(5):36. doi: 10.1186/scrt36 (PMC3025438; doi:10.1186/scrt36)
Supplement: Additional file 2 — Table S1. Dopamine clearance rates in vivo. [file scrt36-S2.PDF]

Supplementary Table 2 – Grammatopoulos et al., 2010

Table S2. Dopamine clearance rates *in vivo*.

Mouse	DA Clearance Rate (μM/s)		DA Clearance Rate (% change)
	C17.mock	C17.hDAT	C17.hDAT vs C17.mock
#1	0.0374	0.0488	30% increase
#2	0.0698	0.0829	19% increase
#3	0.0328	0.0510	55% increase
#4	0.0419	0.0622	48% increase
#5	0.0427	0.0278	35% decrease

DA clearance was measured in brain slices from 5 mice, each with a C17.mock graft in the right cerebral cortex and a C17.hDAT cell graft in the left cerebral cortex. Pressure-ejection of DA was adjusted for each recording site so that the maximum amplitudes (Amax) of the DA signals were similar in the paired recordings performed in each mouse and between experiments in the five mice. Amax values: C17.mock graft = 2.21 ± 0.05 μM and C17.hDAT graft = 2.07 ± 0.14 μM (n = 5).

In 4/5 mice the paired recording results showed faster clearance rates in the track containing the grafted C17.hDAT cells than in the track containing the grafted C17.mock cells, but in the fifth mouse the opposite relationship was observed.

In the first four mice, the mean increase in clearance rate of the C17.hDAT vs C17.mock cells was 38.0 ± 8.2%, but for the fifth mouse a 35% decrease in clearance rate was observed. Since we have no reason to exclude the fifth mouse, our overall results revealed no statistically significant differences.

DA: dopamine; C17.hDAT: C17.2 neural stem cell line stably expressing a construct containing the hDAT sequence; C17.mock: C17.2 neural stem cell line stably expressing a construct lacking the hDAT sequence.
