# Supplementary material for: Neurotransplantation of stem cells genetically modified to express human dopamine transporter reduces alcohol consumption
Source: Stem Cell Res Ther. 2010 Dec 1;1(5):36. doi: 10.1186/scrt36 (PMC3025438; doi:10.1186/scrt36)
Supplement: Additional file 3 — Figure S2. DA clearance parameters measured in C17.mock and C17.hDAT stem cells transplanted into mouse cerebral cortex. [file scrt36-S3.PDF]

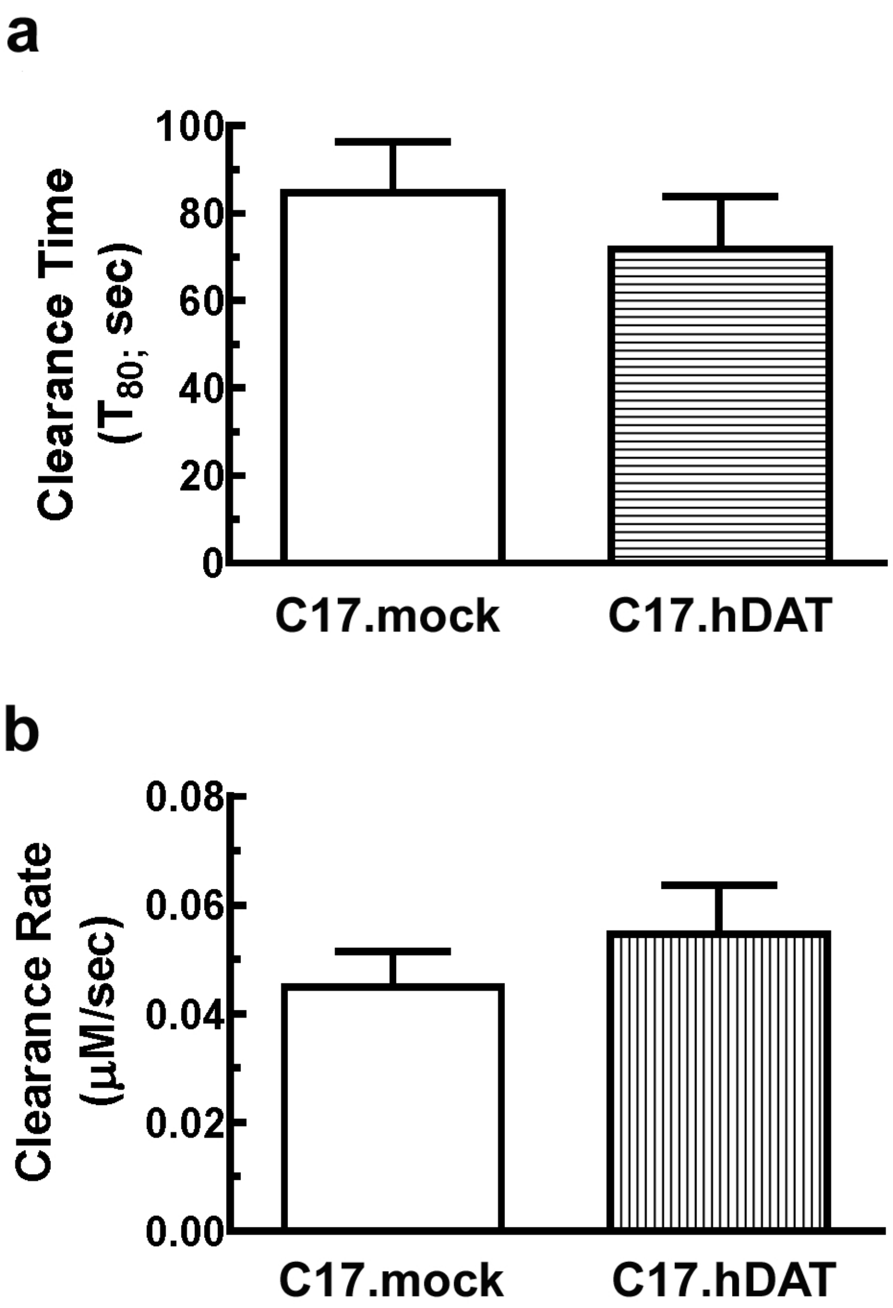

Figure S2

DA clearance parameters measured in C17.mock and C17.hDAT stem cells transplanted into mouse cerebral cortex. High speed chronoamperometry was used to measure the clearance of locally-applied DA in brain slices along the tracks where stem cells were implanted. (a) Clearance time. Local application of DA (C17.mock: 7.6 ± 1.5 psi-sec; C17.hDAT: 6.2 ± 1.8; n = 5) resulted in signals with similar maximal amplitudes in the transplanted C17.mock (Amax: 2.21 ± 0.05 µM) and C17.hDAT (2.07 ± 0.14) cells. As would be expected in the cells with active DATs, the clearance times (T80) were lower in 4/5 C17.hDAT transplants, as compared to the respective C17.mock control transplant in the same mouse. However, these reductions varied widely from 4% to 48% of control. Overall, the mean T80 value was reduced by 15% in the C17.hDAT transplants (C17.mock: 84.8 ± 11.6 sec; C17.hDAT: 71.9 ± 12), but this trend was not statistically significant. (b) Clearance rate. T80 is defined as the time that it takes the DA signal to rise to its maximum and to decay by 80%. Clearance rates are derived from the linear portion of the decaying DA signals. Mean values ± SEM are shown for n = 5/group.
